# Safety and Ergonomic Challenges of Ventilating a Premature Infant During Delayed Cord Clamping

**DOI:** 10.3390/children6040059

**Published:** 2019-04-13

**Authors:** Wannasiri Lapcharoensap, Allison Cong, Jules Sherman, Doug Schwandt, Susan Crowe, Kay Daniels, Henry C. Lee

**Affiliations:** 1 Division of Neonatology, Department of Pediatrics, Oregon Health & Science University, Portland, OR 97239, USA; 2 Department of Pediatrics, Stanford University, Palo Alto, CA 94304, USA; acong@stanford.edu (A.C.); julessherman@alumni.stanford.edu (J.S.); doug.schwandt@gmail.com (D.S.); hclee@stanford.edu (H.C.L.); 3 Department of Obstetrics and Gynecology, Stanford University, Palo Alto, CA 94304, USA; scrowe@stanford.edu (S.C.); kdaniels@stanford.edu (K.D.); 4 California Perinatal Quality Care Collaborative, Palo Alto, CA 94305, USA

**Keywords:** neonatology, resuscitation, delivery room, delayed cord clamping, premature infants, simulation

## Abstract

Delayed cord clamping (DCC) is endorsed by multiple professional organizations for both term and preterm infants. In preterm infants, DCC has been shown to reduce intraventricular hemorrhage, lower incidence of necrotizing enterocolitis, and reduce the need for transfusions. Furthermore, in preterm animal models, ventilation during DCC leads to improved hemodynamics. While providing ventilation and continuous positive airway pressure (CPAP) during DCC may benefit infants, the logistics of performing such a maneuver can be complicated. In this simulation-based study, we sought to explore attitudes of providers along with the safety and ergonomic challenges involved with safely resuscitating a newborn infant while attached to the placenta. Multidisciplinary workshops were held simulating vaginal and Caesarean deliveries, during which providers started positive pressure ventilation and transitioned to holding CPAP on a preterm manikin. Review of videos identified 5 themes of concerns: sterility, equipment, mobility, space and workflow, and communication. In this study, simulation was a key methodology for safe identification of various safety and ergonomic issues related to implementation of ventilation during DCC. Centers interested in implementing DCC with ventilation are encouraged to form multidisciplinary work groups and utilize simulations prior to performing care on infants.

## 1. Introduction

In recent years, the practice of delayed cord clamping (DCC), defined as clamping of the umbilical cord 30 s or more after delivery, has been endorsed by multiple professional organizations (Table 1). Current evidence suggests that DCC may improve outcomes by allowing for greater volume of blood transferred from the placenta to the infant [1]. DCC compared to immediate cord clamping aids in the physiological transition from fetus to newborn, and is correlated with favorable outcomes (Table 2). Systematic reviews of randomized controlled trials in term and preterm infants have suggested that after delayed clamping, neonates showed signs of improved circulation, increased hematocrit levels, lower incidence of necrotizing enterocolitis or intraventricular hemorrhage, and decreased hospital mortality [2,3,4,5]. Other studies have discussed long term effects, such as a reduction in the risk of newborn anemia and need for transfusion due to improved iron status, measured by ferritin and stored iron concentration. However, both the American Academy of Pediatrics (AAP) and the American College of Obstetricians and Gynecologists (ACOG) draw caution to DCC and advise for individualized care in situations involving abnormal placentation, including placental abruption, placenta previa, umbilical cord avulsion, as well as situations requiring immediate neonatal resuscitation at birth. 

Experimental studies in lamb models have demonstrated that clamping of the cord prior to ventilation leads to unstable hemodynamic parameters, while ventilation before clamping leads to higher systemic and cerebral oxygen saturation [15,16,17]. Thus, evidence from animal models would suggest that inflation of the lung prior to cord clamping is critical. Putting these two practices together, a single center study reported that premature infants who started breathing spontaneously during DCC had improved outcomes compared to those who did not breathe spontaneously [18]. Furthermore, even in spontaneously breathing infants, there is increasing evidence that in those premature infants born less than 28 weeks gestational age, continuous positive airway pressure (CPAP) immediately after birth improves symptoms of respiratory distress syndrome, reduces the need for intubation and exogenous surfactant, and may reduce bronchopulmonary dysplasia [19,20,21,22,23]. Therefore, it is hypothesized that the ability to apply CPAP and ventilate an infant while attached to the umbilical cord may confer benefits to the premature infant and improve outcomes. While challenging, the process of establishing ventilation in premature infants during delayed cord clamping has been reported as a feasible practice [24,25,26,27], however there is scarce detail on what practical challenges may be involved during implementation.

In this simulation study, we sought to explore attitudes of providers, as well as the safety and ergonomic challenges of how providers may safely resuscitate and apply CPAP to an infant while still attached to the placenta via the umbilical cord.

## 2. Materials and Methods

### 2.1. Participants

Healthcare professionals from neonatal and obstetric (OB) teams were recruited via electronic flyers to participate in a 3-hour simulation workshop exploring different configurations for delivering CPAP to a premature infant during vaginal and operative deliveries. Providers routinely participate in deliveries at hospitals with a level IV neonatal intensive care unit (NICU)—either Lucile Packard Children’s Hospital at Stanford University or University of California San Francisco Benioff Children’s Hospital. A level IV NICU is defined as a regional ICU with a full range of pediatric medical and surgical subspecialists [28]. At the time of the study, neither center was routinely performing delayed cord clamping on premature infants. This study was approved by the Institutional Review Board at Stanford University and informed written consent was obtained from all subjects.

### 2.2. Setting and Design

**Figure 1:**
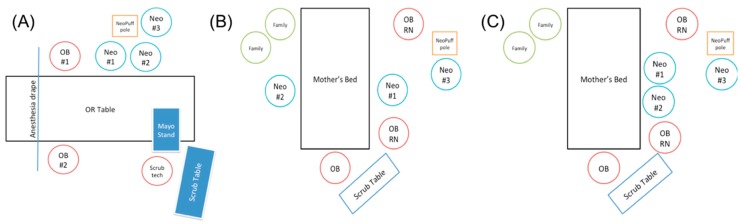
Room layout for simulations of (**A**) C-section delivery and (**B**,**C**) vaginal deliveries. In the vaginal delivery room setup, neonatal providers could choose to stand on opposite sides of the delivery bed (**B**) or the same side (**C**). Neo = neonatal team member, OB RN = obstetric nurse, OB = obstetrician.

**Figure 2:**
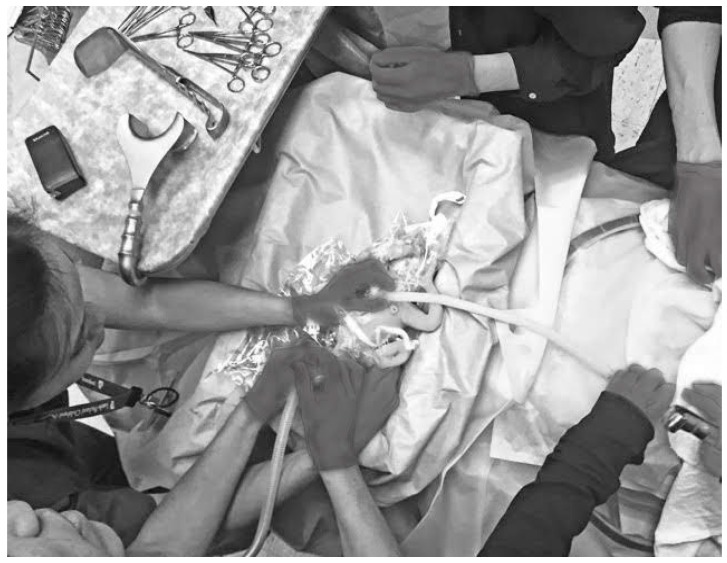
Simulation of delayed cord clamping with a premature manikin. In this image, a member of the neonatal team is assessing the infant’s heart rate, while another neonatal provider is providing ventilation with a facemask and t-piece resuscitator.

All simulated resuscitation scenarios were performed at the Center for Advanced Pediatric and Perinatal Education (CAPE). CAPE is a simulation-based training and research center equipped with cameras and microphones for accurate review of the scenarios. The simulation center was set-up as an operating room suite for Cesarean sections (C-section) in one area and vaginal deliveries in a different area (Figure 1). Each session had a mix of attendance, and when there were not enough people, volunteers from the simulation center helped stand-in the various positions. Providers were instructed to resuscitate a premature manikin (27 weeks gestational age, approximate weight 1 kilogram) attached to the umbilical cord (63 centimeters long), while utilizing a mock-up cardboard box simulating a NeoPuff ™ (Fisher and Paykel Healthcare, Irvine, CA, USA) with a t-piece resuscitator attached to a rolling intravenous (IV) pole (Figure 2). In terms of the umbilical cord, a participant was often holding the cord down at the end “attached” to the placenta. Therefore, the functional length of the umbilical cord in our scenarios was likely closer to the published average length of a term umbilical cord, which is 56.6 centimeters (cm), with an interquartile range of 48 to 63 cm [29].

The scenario included delivery of the infant who had an initial heart rate of 80/minute responsive to positive pressure ventilation (PPV). Providers were instructed to switch to holding CPAP on the baby’s face after PPV. No intubation or chest compressions were included as part of the scenarios. The cord was clamped after 60 s. Each session was conducted similar to a workshop and subjects provided feedback as the session progressed.

Video and audio recordings were captured during each session and transcribed. Transcriptions were then analyzed using qualitative methods to identify common themes related to ergonomic challenges and safety issues for the mother and infant. 

## 3. Results

There was a total of 5 sessions with 19 participants, representing a broad multidisciplinary group. The participants included: 2 neonatal fellows, 3 pediatricians, 2 neonatal nurse practitioners, 3 attending neonatologists, 1 neonatal respiratory therapist, 3 neonatal nurses, 4 obstetricians, and 1 family advocate. Each scenario included a full complement of the healthcare team to perform neonatal resuscitation for a preterm infant. During these sessions, we successfully identified significant safety and ergonomic challenges and divided these into 5 key themes (Table 3). With the exception of theme 1, all themes were applicable to both the C-section and vaginal delivery set-up.

### 3.1. Theme 1: Sterility

As many deliveries are done via C-section, the first question many had involved the availability of sterile equipment and whether personnel needed to be sterile: *“In the C-section delivery, the difficulty is the sterile factor”, “…it gets so close to the mom’s incision”, “Anything that is within 12 inches of the incision needs to be sterile.”* In particular, at the time of the simulations, a sterile neonatal mask was not available on the market: *“If you can get that one piece sterile, that would solve a lot of problems.”*

The need to maintain sterility to decrease infections also would increase personnel numbers: *“We need a nonsterile person to control the NeoPuff”, “… that means you would need to have two sterile people and 1 nonsterile person so 3 people for all deliveries.”* Many expressed that this may be challenging in community hospitals or hospitals with lower volumes.

### 3.2. Theme 2: Equipment

While maintaining sterility may require small modifications to current equipment, one of the challenges of holding CPAP is the need for a flat surface to optimize a mask hold on the infant. Though surfaces have been developed, providers encounter difficulties reaching the surface due to limitations of the umbilical cord. *“This is going to be a problem because I can’t reach the baby with this (equipment)”, “The cord is really short”, “If we are going to be doing delayed cord clamping, we need to get new equipment.”*

### 3.3. Theme 3: Mobility

Once the umbilical cord was clamped, providers simulated the transfer of the infant to a radiant warmer. Questions arose on how to maintain functional residual capacity with good CPAP during this time: *“how will we move the baby with CPAP”, “it’s probably nicer to have the baby transferred on a bed or flat surface”.* Another difficulty observed was that the infant is transferred several times within the first hour of life: *“why are we doing all these transfers? … You would not do 3 transfers on any other critically ill patients, why do it for preemies?... they are at risk for (intraventricular hemorrhage).”*

### 3.4. Theme 4: Space and Workflow

In order to achieve the goal of providing CPAP during deliveries, many providers were working closely together in a small space. *“There isn’t very much space.”* The teams had to creatively figure out ideal arrangements around the mother’s bed or operating table in order to appropriately and safely care for the infant and mother simultaneously. *“Now that we want to do CPAP immediately, that changes everything”, “Well, we would have to redo our (delivery) room teams”.* Teams also had to discuss each other’s responsibilities prior to starting: *“I think we cannot depend on the OB… to help out with stimulating the baby”.*

### 3.5. Theme 5: Communication

Obstetric and neonatal providers all voiced concerns about ensuring the safety of their patient, highlighting the importance of communication between teams. *“Communication between anesthesiologist and OB and the pediatrician team is very important”, “in my experience, everyone has to be on the same page in order to do delayed cord clamping”.* In particular, when there are safety concerns, communication prior to and after delivery are imperative, so that the cord can be clamped immediately if needed: *“there’s a lot of situations where you wouldn’t do this”.*

However, many also viewed this as an opportunity to improve collaboration between disciplines: *“I think it’s going to influence our culture across the board as we make these changes… who would ever think… that we would be together at the operating table. so I just think this is going to influence really broadly”, “I think this could influence our culture of safety, our ability to know each other… knowing who is there increases our culture of safety and communication is better”, “and it’s been shown that optimizing communication improves safety, so I think that this could really have a broad impact on a lot of things.”*

## 4. Discussion

Simulation was a key methodology in order to identify various safety and ergonomic issues in carrying out new guidelines and recommendations surrounding DCC. The ACOG statement endorsing DCC specifically states: “Communication with the neonatal care provider is essential”, highlighting the need for effective interdisciplinary workflow. Our series of simulations affirmed this statement. While sterility was a main concern in the C-section delivery setup, the issues of equipment availability, mobility, space and workflow, and communication were present in both the C-section and vaginal deliveries. We found that the use of simulation workshops could also uncover safety issues that are ongoing. The theme of mobility, i.e., repeatedly transferring a premature infant, can also be applicable to all deliveries, including those that do not involve delayed cord clamping and may be institution-specific. The practice at some institutions include transfer of an infant directly to an incubator after delivery, which will then be taken to the NICU; thus, limiting the number of transfers [30].

A few of the themes (space and workflow, communication, and equipment) could potentially be addressed with real life experience and repeated exposures. In a recent study, 16% of infants randomized to DCC violated protocol because the umbilical cord was too short [24]. However, the authors noted that this issue improved with experience, likely with increased familiarity with available equipment and workflow. Key challenges with workflow and communication can also be addressed with implementation of protocols, along with dissemination of information to involved parties (OB, neonatal, anesthesia providers, and staff). Other challenges could involve industry, such as development of a resuscitation surface that would allow all infants to receive appropriate ventilation no matter the umbilical cord length, or re-packaging items, such as a t-piece resuscitator and infant mask, in sterile packaging. 

Other investigators have discovered that considering similar issues, such as preparation, crowding, sterility, and equipment for thermoregulation, to be key components of optimizing DCC and early resuscitation [25,27,31]. There are opportunities to learn how to optimize care for the infant-mother dyad while carrying out DCC and providing CPAP to infants <28 weeks gestational age. Careful consideration of processes prior to adoption of new guidelines are important, particularly when it is not absolutely certain that it will confer greater benefit to the infant compared to current standards of DCC alone. A recently published single-center study of premature infants receiving DCC compared infants that received ventilation (PPV, CPAP) to those that received stimulation showed no differences in measurable outcomes [26]. In that study, however, 90%–92% of infants were spontaneously breathing by 1 min after birth in either the DCC alone or ventilation with DCC group. It remains unclear if ventilation during DCC may specifically benefit those infants who do not spontaneously start breathing. Based on impressive work from animal models, starting positive pressure ventilation while attached to the cord may allow the infant to have an easier transition to extrauterine life if spontaneous breathing does not occur [15,17]. At time of publication submission, clinicaltrials.gov lists several ongoing trials in the United States, Canada, and Italy to elucidate whether CPAP during DCC results in decreased morbidities and improves long term outcomes, including one study with a completed pilot study. 

The strengths of this study included the use of a multidisciplinary team, including a family member, the use of video-taping to review workshops that were held, and the ability to safely try out different methods of CPAP delivery. Limitations included the fact that it was done at a single center, scenarios were repetitive, utilizing the same set-up, and discussion time was limited. In addition, while using simulation as a methodology is an ideal manner to explore safety issues, it does not address other clinical considerations, such as actual admission temperature for the premature infant, ability to assess color change on carbon dioxide detector, or ability to adequately assess infant’s heart rate. Further work in simulation may be able to test whether altering the workflow, personnel, and roles, and adding or modifying equipment, may make the procedure safer and proceed in a smoother fashion. Simulation also provides the opportunity for teams to practice complex clinical scenarios, such as resuscitation during DCC. An important next step for our study would also include exploring scenarios where an infant either does not start spontaneously breathing or does not respond to interventions, as well as videotaping live resuscitations for review.

In conclusion, the delivery of PPV or CPAP with an intact cord is feasible. Optimization of materials, space, and personnel surrounding the mother and infant during the 60 or more seconds of cord attachment after birth will be imperative to safely performing techniques for neonatal resuscitation while remaining attached to the placenta. Creation of protocols developed in a multidisciplinary manner and practice in a simulated, safe environment are keys to successful implementation.

## Figures and Tables

**Table 1:** Current recommendations on umbilical cord clamping from professional organizations.

Organization	Year	Recommendation
World Health Organization (WHO) [6,7]	2006, most recently updated 2017	“In newly-born term or preterm babies who do not require positive-pressure ventilation, the cord should not be clamped earlier than one minute after birth.” “Delayed umbilical cord clamping (not earlier than 1 min after birth) is recommended for improved maternal and infant health and nutrition outcomes.”
International Liaison Committee on Resuscitation (ILCOR) [8]	2010, updated 2015	“DCC for longer than 30 s is reasonable for both term and preterm infants who do not require resuscitation at birth”
Neonatal Resuscitation Program (NRP) guidelines from the American Academy of Pediatrics (AAP) [9]	2017	“Delay in umbilical cord clamping for at least 30–60 s for most vigorous term and preterm infants.”
American College of Obstetricians and Gynecologists (ACOG) [10]	2010, recently updated in 2017	“Delay in umbilical cord clamping in vigorous term and preterm infants for at least 30–60 s after birth”
National Institute for Health and Care Excellence (United Kingdom) [11,12]	2014, updated 2017	“Do not clamp the cord earlier than 1 min from the birth of the baby unless there is concern about the integrity of the cord or the baby has a heart rate below 60 beats/minute that is not getting faster.”
American College of Nurse–Midwives [13]	2014	“For term newborns, delaying the clamping of the cord for 5 min if the newborn is placed skin-to-skin or 2 min with the newborn at or below the level of the introitus ensures the greatest benefit. For preterm newborns, the benefits of delaying cord clamping for 30 to 60 s include a significant reduction in intraventricular hemorrhage and a reduced need for blood transfusion.”
Society of Obstetricians and Gynecologists of Canada [14]	2009, reaffirmed 2018	“Whenever possible, delaying cord clamping by at least 60 s is preferred to clamping earlier in premature newborns (<37 weeks’ gestation) since there is less intraventricular hemorrhage and less need for transfusion in those with late clamping.”

DCC, delayed cord clamping.

**Table 2:** Potential Benefits of delayed cord clamping [2,3,4,5].

**Term Infants**
Increased hemoglobin levels at birth Increased iron stores in first several months of life
**Preterm Infants**
Increased hematocrit levels Reduced need for blood transfusions Reduced incidence of intraventricular hemorrhage Reduced incidence of necrotizing enterocolitis Decreased hospital mortality

**Table 3:** Safety and ergonomic issues identified.

Topic	Identified Challenges
*Sterility*	● Current available respiratory equipment (CPAP mask, ventilation tubing) is not sterile, forcing clinicians to use a nonsterile piece of equipment adjacent to a sterile field with the theoretical risk of increasing surgical site infections. ● An ideal surface would provide adequate warmth to the vulnerable preterm infant. Current commercially available thermal mattresses are not sterile and require a workaround. ● Maintaining sterility requires more personnel than community hospitals may be able to staff.
*Equipment*	● CPAP is ideally performed on a flat surface. However, there are limited options on how to best provide CPAP during DCC. Currently there is not an ideal surface and respiratory setup that allows for all infants to receive CPAP during DCC. ● Providers are often limited by the umbilical cord length to reach any available surfaces.
*Mobility*	● Following DCC, the infant needs to be moved from the DCC site to a resuscitation bed or the intensive care unit. Concerns raised about the safety of moving a patient vulnerable to intraventricular hemorrhages multiple times in a short period (DCC to resuscitation bed to the ICU bed). An ideal setup would include minimal transportation and lifting of the infant.
*Space and Workflow*	● In this new arrangement, the workflow was awkward. As there are multiple team members present at the mother’s side in a small space to provide DCC with CPAP, neonatal providers will often start from far away. There needs to be adequate time, space, and communication for the neonatal providers to safely approach the bed.
*Communication*	● Obstetric providers voiced concerns about safety for the mother during DCC and emphasized the need for clear communication between the multidisciplinary teams. ● Neonatal providers request communication about when they are able to approach the mother safely in order to care for the infant. ● There is a need for protocols on when to discontinue DCC due to the infant or maternal status.

CPAP, continuous positive airway pressure; ICU, intensive care unit.
